# Clinicopathological characteristics and treatment outcomes of oesophageal cancer patients in Uganda

**DOI:** 10.3332/ecancer.2023.1576

**Published:** 2023-07-19

**Authors:** Siraji Obayo, Yusuf Mulumba, Cheryl L Thompson, Michael K Gibson, Matthew M Cooney, Jackson Orem

**Affiliations:** 1 Uganda Cancer Institute, Upper Mulago Hill Road PO Box 3935, Kampala, Uganda; 2 Case Western Reserve University, Case Comprehensive Cancer Centre, Cleveland, OH 44106, USA; 3 Case Western Reserve University, University Hospitals Seidman Cancer Center, Cleveland, OH 44106, USA; 4 Vanderbilt University Medical Centre, Vanderbilt-Ingram Cancer Center, Nashville, TN 37232, USA

**Keywords:** oesophageal cancer, clinicopathological characteristics, treatment outcomes, Uganda

## Abstract

**Background:**

Oesophageal cancer is the seventh most common cancer and the sixth leading cause of cancer death worldwide, and its incidence varies globally. In Uganda, the incidence and trend are on the increase. However, there is a paucity of published data regarding this population’s oesophageal cancer clinicopathologic characterisation and treatment outcomes.

**Objectives:**

To study the patients’ clinicopathologic characteristics and treatment outcomes of oesophageal cancer over 10 years at the Uganda Cancer Institute.

**Methods:**

Patients’ charts with histologically confirmed diagnoses of oesophageal cancer for 2009–2019 were identified. Case information, which included patient demographics, history of alcohol use or smoking, tumour location, histological type, tumour grade, clinical TNM (Tumour, Node, Metastasis) staging treatment exposure and treatment outcomes, was evaluated retrospectively. The median survival time was estimated with the Kaplan-Meier method and the median follow-up period was estimated using the reverse Kaplan-Meier.

**Results:**

1,965 oesophageal cancer patients were identified; 1,380(70.23%) were males and 585(29.77 %) females, their mean age was 60.20 years (±12.66). Most males had a history of both alcohol consumption and smoking 640(46.38%). The lower third of the oesophagus was the most common anatomical location 771(39.24%). The majority had squamous cell carcinoma histological type 1,783(90.74%) followed by adenocarcinomas 182(9.26%) in the distal oesophagus. Poorly differentiated tumour grade 743(37.81%) was predominant. The majority of the patients were in stage IVB, 733(37.30%), and most patients were planned for the best supportive care, 731(37.20%). Radiation alone was offered to 621(31.60%) and feeding gastrostomy to 249(12.70%). Treatment outcomes: at the time of the current analysis, 58.68% had died, 1.48% were alive and 39.84% were lost to follow-up. The median follow-up period was 65 months (IQR:35.83–83.30) with a median survival time of 4.47 months (95% CI: 4.17–4.80).

**Conclusion:**

Treatment outcomes of Ugandan oesophageal cancer patients seeking care are poor as most patients present with advanced disease. There is a significant loss of follow-up after treatment initiation. Therefore, reduction in exposure to known modifiable risk factors, early detection and timely referral for treatment strategies are needed to improve outcomes of these patients in our population. Designing interventions to improve treatment adherence is necessary.

## Introduction

Oesophageal cancer is the seventh most common cancer and the sixth leading cause of cancer death worldwide [1]. More than 80% of cases and deaths from oesophageal cancer occur within developing countries [2–4]. The incidence of oesophageal cancer varies globally, with a higher incidence in areas such as Eastern Asia, South Central Asia, South Africa, Eastern Africa and Northern Europe [1–4]. In Uganda, one of the countries comprising the East African sub-region, the incidence of oesophageal cancer is increasing [5–9]. In Uganda, oesophageal cancer ranks sixth and is the third most common cause of cancer-related death, accounting for 8.7% [9]. Treatment of oesophageal cancer depends on the stage of the disease and the patient’s functional status at presentation. Curative treatment usually involves combined modality strategies with surgery, chemotherapy, radiation or endoscopic therapy for mucosal cancers [10–30]. Palliative treatment aims to improve dysphagia, nutrition and quality of life; this can be achieved with radiation, brachytherapy, chemoradiation or endoscopic therapy, which may include dilation and stenting, chemical or ablative debulking, and enteral feeding [10–12, 30–50]. Despite the availability of radiation, chemotherapy, surgical and endoscopic services at Uganda Cancer Institute, a national referral cancer treatment centre and rising oesophageal cancer incidence in Uganda, there is limited data about clinicopathologic features and treatment outcomes for this tumour type. This study aimed to characterise oesophageal cancer patients seeking care over 10 years regarding clinicopathological characteristics and treatment outcomes. Therefore, data obtained from this study will be the first important step to better understand the clinicopathological features and the associated treatment outcomes of oesophageal cancer in Uganda and enhance oesophageal cancer care in our population.

## Methods

This was a retrospective chart review study of confirmed oesophageal cancer patients referred to the Uganda Cancer Institute, a national referral cancer centre, between 2009 and 2019 for treatment. Data collected on each patient’s chart included age, sex, history of alcohol use or smoking, tumour location, histological type, grade, stage, treatment exposures and outcomes. For patients who were no longer in contact with the staff through clinic visits, the patients or their next of kin were contacted through phone calls for patients’ survival status and the dates were recorded. Data were collected and stored using the RedCAP database. This study was approved by the Ugandan National Council for Science and Technology and the Uganda Cancer Institute.

### Statistical analysis

Mean values and SDs were calculated for continuous variables and counts of categorical variables described the distributions of clinicopathologic variables, treatment exposure and outcomes. The relationship between outcomes by stage and treatment was determined by cross-tabulation. The care period at the Uganda Cancer Institute was calculated from the date patients were enrolled to when they were no longer in contact with the clinic or the date of death. The median survival time was estimated with the Kaplan-Meier method and the median follow-up period was estimated using the reverse Kaplan-Meier.

## Results

Over the study period, 1,965 oesophageal patient cases were identified, 1,380(70.23%) were males and 585(29.77%) were females with a mean age of 60.20 years (±12.66). Most males had a history of both alcohol consumption and smoking 640(46.38%), among females majority neither used alcohol nor smoked 363(62.05%). The most common tumour location was the lower third 771(39.24%), followed by the middle third 727(36.99%), then the upper third of the oesophagus 467(23.77%) and the majority had squamous cell histological type 1,783 (90.74%), followed by adenocarcinoma 182(9.26%) in the distal oesophagus. Tumour grades were as follows, poorly differentiated 743(37.81%), moderately differentiated 613(31.20%) and well differentiated 609(30.99%) (Table 1). Staging of the patients, the majority were in stage IVB, 733(37.30%), followed by stage IVA, 421(21.42%), unknown stage 266(13.54%), stage IIIB, 239 (12.16%), then stage IIIA patients 163(8.30%) (Table 1).

Treatment, most got basic supportive care, 731(37.20%), followed by radiation alone 621(31.60%), Feeding gastrostomy alone 249(12.70%) for nutritional purposes then chemoradiation was at 162(8.24%). Survival status of the patients was as follows, dead 1,153(58.68%), followed by those who were lost to follow-up, 783(39.84%), and 29(1.47%) were alive at the closure of this study (Table 2). The median follow-up period was 65 months with an IQR of 35.83–83.30 months and the median survival time after diagnosis with oesophageal cancer was 4.47 months (95% of CI 4.17–4.80).

For most living oesophageal cancer patients, 10(34.48%) were in stage IIA and 6(20.69%) were in stage IIB. The majority of stage IIA and IIB patients had chemoradiation 9(31.03%) followed by transthoracic oesophagectomy plus chemoradiation 4(13.79%), as their treatment. Of those in stage IVA 5(17.24%) who were still alive on chemotherapy alone, 2(6.90%), basic supportive care, 2(6.90%) or had oesophageal stenting, 1(3.44%), had been in care for about 1.7 months by the end of our study. One patent in stage IIIB had gotten oesophageal stenting with no proper rationale from an outside primary healthcare hospital before enrollment (Table 3).

Most dead patients, 676(58.63%), were in stage IVB. Not surprisingly majority had basic supportive care 274(23.76%), palliative radiation 211(18.30%) with 20 Gy in 4 Gy fractions daily, 5 consecutive workdays, feeding gastrostomy 119(10.32%) for nutrition purposes then palliative chemotherapy 45(3.90%), oesophageal stenting 21(1.82%) and chemoradiation 6(0.52%) for their disease (Table S1). One patient in stage IIA had radiation alone due to his advanced age of 88 years to relieve symptoms of dysphagia and chest pain and passed on after 11.93 months. The other in stage IIA was 56 years had chemoradiation and died after 75 months of follow-up, the cause of death was uncertain.

Lost to follow-up patients mostly were in the unknown stage, 259(33.08%), followed by those in stage IIIB 186(23.75%). For those in stage IIIB, the majority had radiation alone, 80(10.22%) then lost to follow-up, while those in the unknown stage most had basic supportive care, 191(24.39%) then lost to follow-up (Table S2).

## Discussion

This study reviewed oesophageal cancer patients’ clinicopathological presentation and treatment outcomes at Uganda’s National Referral Cancer Centre. Out of 1,965 cases reviewed, males were predominant at 1,380(70.23%). The male predominance demonstrated in this study is like other studies performed in Africa, particularly in the Sub-Saharan African region [51, 52]. The male predominance could be explained by the fact that most of the known risk factors for oesophageal cancer are related to behaviour-smoking and excessive alcohol consumption, of which men are known to be worse consumers than women as demonstrated in our study and earlier studies in Africa and China [53–58].

The lower third of the oesophagus was the most frequent anatomical site for oesophageal cancer (39.24%). With (9.26%) being adenocarcinoma and (29.98%) squamous cell carcinoma histological type. This result is consistent with previous studies [59–67], which found a lower third of the oesophagus as the most anatomical site. Our finding differs from other studies [68–73], which reported the middle third of the oesophagus as the most common site for oesophageal cancer. However, we could not establish the reasons for the variation in this anatomical distribution pattern.

Our data demonstrate squamous cell carcinoma is the most common histology representing over >90% of all cases, which is in keeping with other studies from the East African countries of Tanzania, Kenya and Uganda. Southern African countries of Malawi, Mozambique and South Africa. Iran in South Central Asia, and China in East Asia [68, 74–84]. Most tumour grade in our study was poorly differentiated (high grade), keeping with reports from China and Netherlands [72, 85]. This could probably be among the reasons for the high stages in our patients at presentations, as this tumour grade tends to metastasise fast.

In our study, more than 50% of the patients were in stage IV; this finding is consistent with studies from Ethiopia, Zambia, India and the United States of America that reported most of the patients being in stage IV at presentation for care [62, 70, 86–88]. The late-stage presentation and poor performance status are why most of our patients had basic supportive care, among other palliative treatment modalities. 13.54% of the patients in our study had no imaging studies to stage their disease; hence their, stages were unknown, this could have been probably due to the burden of cost in care for the illness, among other reasons in our setting.

Treatment exposure, this study shows most patients had the best supportive care, palliative radiotherapy (20 Gy in 4 Gy fractions) and feeding gastrostomy insertion as their commonest treatments. This is not surprising as the majority of the patients presented for care in the late stage. Our results are in accordance with reports from Mozambique, the Netherlands, the United States of America and Ethiopia, where most patients had the best supportive care, palliative radiotherapy and feeding gastrostomy insertion [81, 85, 89–91].

Treatment outcomes, in our study, death was at 58.68% mostly in stages IVB and IVA, followed by a loss to follow-up patients (39.85%) mostly in the unknown stage and stage IIIB then alive (1.48%) most in stage IIA at the end of this study. This is similar to a study in Brazil that showed death in more than 50% of treated oesophageal cancer patients in advanced stages of the disease [92], as well as other studies from Uganda and the United States of America that showed poor adherence to treatment [93, 94].

A recent Ugandan study reported that depleted financial resources within the first few treatment visits, high transport costs and long distances to the Uganda Cancer Institute hindered patients’ promptness from seeking care and adhering to the treatment schedules. Participants reported that patients would postpone health-seeking visits and miss appointments for their treatments because of a lack of money for transport and upkeep [93].

However, their study was an interview-based qualitative one in which only 16 patients and 17 healthcare professionals were involved and could not establish the magnitudes of associations and characteristics of the participants likely to be associated with the concepts that were being explored.

To overcome transport costs and long-distance travel, the Ugandan government, through the Uganda Cancer Institute, is opening functional regional cancer centers in the East Central (Mbale), Acholi (Gulu), West Nile (Arua) and Southwestern (Mbarara) sub-regions. This will reduce non-adherence to treatment regimens due to long-distance travel costs and consequently improve treatment outcomes.

## Limitations

This study has some limitations. Only a tertiary national referral hospital was involved, thus cases only seen at the regional and district hospitals or self-referred patients seeking treatment outside the country that never reported to the Uganda Cancer Institute for care may have been missed, and thus case ascertainment may be incomplete. We cannot exclude diagnostic bias based on interest, expertise, and access to diagnostic facilities. Lost to follow-up patients whose phones were no longer on the network, and we could not confirm their actual vital status that may have been missed, thus underestimating their treatment outcomes.

## Conclusion

This study confirmed the clinicopathological characteristics and treatment outcomes data of oesophageal cancer in Uganda, which validates the predominance of high-grade oesophageal squamous cell carcinoma with the late presentation, poor treatment outcomes and significant loss of follow-up in this context. This study demonstrates the need to prioritise oesophageal cancer on the national health agenda, including promoting preventative strategies, cost-effective early assessment, detection and timely treatment and designing interventions to improve treatment adherence such as using appointment reminders via mobile phones and other technologies, enhancing referral pathways and patient navigation is necessary.

## Conflicts of interest

The authors declare that there is no conflict of interest.

## Funding

Research support from the Conquer Cancer Foundation of the American Society of Clinical Oncology as part of the Long-term International Fellowship (LIFe) Award.

## Figures and Tables

**Table S1.** Oesophageal cancer patients enrolled in care who died.

Stage	Treatment									
	RT	BSC	FG	CM	CRT	STENTING	T.ESO	T.ESO+RT	CRT+T.ESO	*N* (%)
IIA	1	-	-	-	1	-	-	-	-	2(0.17)
IIB		-	-	-	1	-	-	-	-	1(0.09)
IIIA	2	-	-	3	6	-	-	-	-	11(0.95)
IIIB	16	-	2	7	20	2	-	-	2	49(4.25)
IIIC	6	-	-	1	4	-	2	1	-	14(1.21)
IVA	165	113	73	18	17	7	-	-	-	393(34.08)
IVB	211	274	119	45	6	21	-	-	-	676(58.63)
Unknown	1	4	1	1	-	-	-	-	-	7(0.61)
Total* N* (%)	402	391	195	75	55	30	2	1	2	1153(100)

RT = Radiation, BSC = Basic supportive care, FG = Feeding gastrostomy, CM = Chemotherapy, CRT = Chemoradiation, T.ESO = Transthoracic oesophagectomy

**Table S2.** Oesophageal cancer patients who were lost to follow-up by stage and treatment.

Stage	Treatment										
	BSC	RT	FG	CRT	CM	STENTING	T.ESO+CRT	T.ESO	T.ESO+CM	T.ESO+RT	*N* (%)
IIA	15	11	1	9	3	-	1	2	-	-	42(5.36)
IIB	7	4	-	15	-	-	-	-	-	-	26(3.32)
IIIA	40	53	3	31	14	2	2	-	2	1	148(18.9)
IIIB	45	80	5	32	17	6	-	-	-	1	186(23.75)
IIIC	5	28	1	5	1	2	-	-	-	-	42(5.36)
IVA	7	2	5	-	3	6	-	-	-	-	23(2.94)
IVB	28	6	13	2	5	3	-	-	-	-	57(7.28)
Unknown	191	35	26	-	6	1	-	-	-	-	259(33.08)
Total *N* (%)	338	219	54	94	49	20	3	2	2	2	783(100)

BSC = Basic supportive care, RT = Radiation, FG = Feeding gastrostomy, CRT = Chemoradiation, CM = Chemotherapy, T.ESO = Transthoracic oesophagectomy

**Table 1.** Clinicopathologic characteristics of oesophageal cancer patients enrolled into care.

Characteristic	Mean(SD)	Number	Proportion (%)
Sex			
Male		1,380	70.23
Female		585	29.77
Age	60.20(12.66)		
≤50		464	23.61
>50		1,501	76.39
Males’ history of alcohol use or smoking			
Alcohol only		308	22.32
Smoking only		55	3.98
Alcohol and smoking		640	46.38
None users		377	27.32
Females’ history of alcohol use or smoking			
Alcohol only		125	21.37
Smoking only		18	3.08
Alcohol and smoking		79	13.5
None users		363	62.05
Tumour location			
Lower third of oesophagus		771	39.24
Mid third of oesophagus		727	36.99
Upper third of oesophagus		467	23.77
Histological type			
Squamous cell carcinoma		1783	90.74
Adenocarcinoma		182	9.26
Tumour grade			
Poorly differentiated		743	37.81
Moderately differentiated		613	31.2
Well differentiated		609	30.99
Clinical TNM stage			
IIA		54	2.75
IIB		33	1.68
IIIA		163	8.3
IIIB		239	12.16
IIIC		56	2.85
IVA		421	21.42
IVB		733	37.3
Unknown		266	13.54

SD = Standard deviation

**Table 2.** Treatment and outcomes of oesophageal cancer patients enrolled into care.

Treatment	Number	Proportion (%)
Basic supportive care	731	37.2
Radiation alone	621	31.6
Feeding gastrostomy alone	249	12.7
Chemoradiation	162	8.24
Chemotherapy alone	127	6.46
Oesophageal stenting	52	2.64
Transthoracic oesophagectomy + Chemoradiation	7	0.36
Transthoracic oesophagectomy	4	0.2
Transthoracic oesophagectomy **+** Chemotherapy	4	0.2
Transthoracic oesophagectomy **+** Radiation	3	0.15
Chemoradiation + Transthoracic oesophagectomy	5	0.25
Survival status		
Dead	1,153	58.68
Lost to follow-up	783	39.84
Alive	29	1.48

**Table 3.** Oesophageal cancer patients in care who were still alive by stage and treatment.

Stage	Treatment							
	CRT	T.ESO +CRT	CM	CRT +T.ESO	T.ESO +CM	BSC	STENTING	*N* (%)
IIA	5	3	-	-	2	-	-	10(34.48)
IIB	4	1	-	1	-	-	-	6(20.69)
IIIA	3	-	1	-	-	-	-	4(13.79)
IIIB	1	-		2	-	-	1	4(13.79)
IVA	-	-	2	-	-	2	1	5(17.24)
Total (*N* %)	13(44.83)	4(13.79)	3(10.34)	3(10.34)	2(6.9)	2(6.9)	2(6.9)	29(100.00)

CRT = Chemoradiation, T.ESO = Transthoracic oesophagectomy, CM = Chemotherapy, BSC = Basic supportive care

## References

[1] Sung H, Ferlay J, Siegel RL (2021). GLOBOCAN estimates of incidence and mortality worldwide for 36 cancers in 185 countries. CA Cancer J Clin.

[2] Ferlay J, Soerjomataram I, Dikshit R (2015). Cancer incidence and mortality worldwide. Int J Cancer.

[3] Wong MCS, Hamilton W, Whiteman DC (2018). Global incidence and mortality of oesophageal cancer and their correlation with socioeconomic indicators temporal patterns and trends in 41 countries. Sci Rep.

[4] Cheng ML, Zhang L, Borok M (2015). The incidence of oesophageal cancer in Eastern Africa: identification of a new geographic hot spot?. Cancer Epidemiol.

[5] Obayo S, Luswa L, Orem J (2017). Gastrointestinal malignancies at five regional referral hospitals in Uganda. Afri Health Sci.

[6] Wabinga H, Nambooze S, Amulen PM (2014). Trends in the incidence of cancer in Kampala, Uganda1991-2010. Int J Cancer.

[7] GBD 2017 Oesophageal Cancer Collaborators (2020). The global, regional, and national burden of oesophageal cancer and its attributable risk factors in 195 countries and territories, 1990-2017: a systematic analysis for the Global Burden of Disease Study 2017. Lancet Gastroenterol Hepatol.

[8] Huang J, Koulaouzidis A, Marlicz W (2021). Global burden, risk factors, and trends of oesophageal cancer: an analysis of cancer registries from 48 countries. Cancer.

[9] Ferlay J, Ervik M, Lam F (2020). Global Cancer Observatory: Cancer Today.

[10] Evans JA, Early DS, Chandraskhara V (2013). The role of endoscopy in the assessment and treatment of oesophageal cancer. Gastrointest Endosc.

[11] Asombang AW, Chishinga N, Nkhoma A (2019). Systematic review and meta-analysis of oesophageal cancer in Africa: epidemiology, risk factors, management and outcomes. World J Gastroenterol.

[12] Ancona E, Ruol A, Santi S (2001). Only pathologic complete response to neoadjuvant chemotherapy improves significantly the long term survival of patients with resectable oesophageal squamous cell carcinoma: final report of a randomized, controlled trial of preoperative chemotherapy versus surgery alone. Cancer.

[13] Medical Research Council Oesophageal Cancer Working Group (2002). Surgical resection with or without preoperative chemotherapy in oesophageal cancer: a randomised controlled trial. Lancet.

[14] Ando N, Iizuka T, Ide H (2003). Surgery plus chemotherapy compared with surgery alone for localized squamous cell carcinoma of the thoracic oesophagus: a Japan clinical oncology group study. J Clin Oncol.

[15] Urschel JD, Vasan H (2003). A meta-analysis of randomized controlled trials that compared neoadjuvant chemoradiation and surgery to surgery alone for resectable oesophageal cancer. Am J Surg.

[16] Malthaner RA, Wong RK, Rumble RB (2004). Neoadjuvant or adjuvant therapy for resectable oesophageal cancer: a systematic review and meta-analysis. BMC Med.

[17] Gebski V, Burmeister B, Smithers BM (2007). Survival benefits from neoadjuvant chemoradiotherapy or chemotherapy in oesophageal carcinoma: a meta-analysis. Lancet Oncol.

[18] Pennathur A, Luketich JD, Landreneau RJ (2008). Long-term results of a phase II trial of neoadjuvant chemotherapy followed by oesophagectomy for locally advanced oesophageal neoplasm. Ann Thorac Surg.

[19] Pennathur A, Luketich JD (2008). Resection for oesophageal cancer: strategies for optimal management. Ann Thorac Surg.

[20] Tepper J, Krasna MJ, Niedzwiecki D (2008). Phase III trial of trimodality therapy with cisplatin, fluorouracil, radiotherapy, and surgery compared with surgery alone for oesophageal cancer. J Clin Oncol.

[21] Pennathur A, Farkas A, Krasinskas AM (2009). Oesophagectomy for T1 oesophageal cancer: outcomes in 100 patients and implications for endoscopic therapy. Ann Thorac Surg.

[22] Jin HL, Zhu H, Ling TS (2009). Neoadjuvant chemoradiotherapy for resectable oesophageal carcinoma: a meta-analysis. World J Gastroenterol.

[23] Lv J, Cao XF, Zhu B (2010). Long-term efficacy of perioperative chemoradiotherapy on oesophageal squamous cell carcinoma. World J Gastroenterol.

[24] Sjoquist KM, Burmeister BH, Smithers BM (2011). Survival after neoadjuvant chemotherapy or chemoradiotherapy for resectable oesophageal carcinoma: an updated meta-analysis. Lancet Oncol.

[25] Ando N, Kato H, Igaki H (2012). A randomized trial comparing postoperative adjuvant chemotherapy with cisplatin and 5-fluorouracil versus preoperative chemotherapy for localized advanced squamous cell carcinoma of the thoracic oesophagus. Ann Surg Oncol.

[26] Van Hagen P, Hulshof MC, Van Lanschot JJ (2012). Preoperative chemoradiotherapy for oesophageal or junctional cancer. N Engl J Med.

[27] Almhanna K, Strosberg JR (2012). Multimodality approach for locally advanced oesophageal cancer. World J Gastroenterol.

[28] Vijayakumar M, Burrah R, Hari K (2013). Oesophagectomy for cancer of the oesophagus. A regional cancer centre experience. Indian J Surg Oncol.

[29] Kidane B, Coughlin S, Vogt K (2015). Preoperative chemotherapy for resectable thoracic oesophageal cancer. Cochrane Database Syst Rev.

[30] Weidenbaum C, Gibson MK (2022). Approach to localized squamous cell cancer of the oesophagus. Curr Treat Options Oncol.

[31] Luketich JD, Christie NA, Buenaventura PO (2000). Endoscopic photodynamic therapy for obstructing oesophageal cancer: 77 cases over a 2-year period. Surg Endosc.

[32] Litle VR, Luketich JD, Christie NA (2003). Photodynamic therapy as palliation for oesophageal cancer: experience in 215 patients. Ann Thorac Surg.

[33] Petersen BT, Chuttani R, Croffie J (2006). Photodynamic therapy for gastrointestinal disease. Gastrointest Endosc.

[34] Ndonga AK, Rucha MR, Oigara R (2008). Oesophageal cancer and experience with endoscopic stent intubation at St. Mary’s Hospital, Nairobi. Ann Afr Surg.

[35] Shenfine J, McNamee P, Steen N (2009). A randomized controlled clinical trial of palliative therapies for patients with inoperable oesophageal cancer. Am J Gastroenterol.

[36] Diamantis G, Scarpa M, Bocus P (2011). Quality of life in patients with oesophageal stenting for the palliation of malignant dysphagia. World J Gastroenterol.

[37] Thumbs A, Borgstein E, Vigna L (2012). Self-expanding metal stents (SEMS) for patients with advanced oesophageal cancer in Malawi: an effective palliative treatment. J Surg Oncol.

[38] Jiang XJ, Song MQ, Xin YN (2012). Endoscopic stenting and concurrent chemoradiotherapy for advanced oesophageal cancer: a case-control study. World J Gastroenterol.

[39] Rueth NM, Shaw D, D'Cunha J (2012). Oesophageal stenting and radiotherapy: a multimodal approach for the palliation of symptomatic malignant dysphagia. Ann Surg Oncol.

[40] Yang C, Li J, Fu W (2014). Interventions for dysphagia in oesophageal cancer. Cochrane Database Syst Rev.

[41] Parthipun A, Diamantopoulos A, Shaw A (2014). Self-expanding metal stents in palliative malignant oesophageal dysplasia. Ann Palliat Med.

[42] Altuntaş B, Yekeler E, Subaşı M (2015). Palliation of malignant dysphagia with self expandable oesophageal stents. Turk Gogus Kalp Dama.

[43] Ramakrishnaiah VPN, Malage S, Sreenath GS (2016). Palliation of dysphagia in carcinoma oesophagus. Clin Med Insights Gastroenterol.

[44] Bhatnagar AR, Bhandari R, Kumbhaj P (2017). Role of palliative radiotherapy, chemotherapy and stents for dysphagia and quality of life improvement in advanced oesophageal cancer. The optimal management?. Ann Oncol.

[45] Kim KY, Tsauo J, Song HY (2017). Self-expandable metallic stent placement for the palliation of oesophageal cancer. J Korean Med Sci.

[46] Włodarczyk JR, Kuzdzał J (2018). Stenting in palliation of unresectable oesophageal cancer. World J Surg.

[47] Buckle GC, Mahapatra R, Mwachiro M (2021). Optimal management of oesophageal cancer in Africa: a systemic review of treatment strategies. Int J Cancer.

[48] Birla R, Achim F, Marica C (2020). Palliation of dysphagia in patients with unresectable oesophageal tumours-current methods and indications-a review. Dig Med Res.

[49] Koggel LM, Lantinga MA, Siersema PD (2021). Palliation of malignant dysphagia: stent or radiotherapy. Ann Oesophagus.

[50] Mwachiro M, Parker R, Lando J (2022). Predictors of adverse events and early mortality after oesophageal stent placement in a low resource setting: a series of 3823 patients in Kenya. Endosc Int Open.

[51] Middleton DRS, Bouaoun L, Hanisch R (2018). Oesophageal cancer male to female incidence ratios in Africa: a systematic review and meta-analysis of geographic, time and age trends. Cancer Epidemiol.

[52] Kachala R (2010). Systematic review: epidemiology of oesophageal cancer in Sub Saharan Africa. Malawi Med J.

[53] Pampel F (2008). Tobacco use in Sub-Sahara Africa estimates from the demographic health surveys. Soc Sci Med.

[54] Middleton DRS, Mmbaga BT, Menya D (2022). Alcohol consumption and oesophageal squamous cell cancer risk in East Africa: finding from the large multicenter ESCCAPE case-control study in Kenya, Tanzania, and Malawi. Lancet Glob Health.

[55] Li Q, Hsia J, Yang G (2011). Prevalence of smoking in China in 2010. N Engl J Med.

[56] Millwood IY, Li L, Smith M (2013). Alcohol consumption in 0.5 million people from 10 diverse regions of China: prevalence, patterns and socio-demographic and health-related correlates. Int J Epidemiol.

[57] Liu S, Zhang M, Yang L (2017). Prevalence and patterns of tobacco smoking among Chinese adult men and women findings of the 2010 national smoking survey. J Epidemiol Community Health.

[58] Yang X, Chen X, Zhuang M (2017). Smoking and alcohol drinking in relation to the risk of oesophageal squamous cell carcinoma: a population-based case-control study in China. Sci Rep.

[59] Wakhisi J, Patel K, Buziba N (2005). Oesophageal cancer in north rift valley of western Kenya. Afr Health Sci.

[60] Deybasso HA, Roba KT, Nega B (2021). Clinico-pathological findings and spatial distributions of oesophageal cancer in Arsi Zone, Central Ethiopia. Cancer Manag Res.

[61] Chetwood JD, Finch PJ, Kankwatira A (2018). Five-year single-centre experience of carcinoma of the oesophagus from Blantyre, Malawi. BMJ Open Gastro.

[62] Hassen HY, Teka MA, Addisse A (2021). Survival status of oesophageal cancer patients and its determinants in Ethiopia: a facility based retrospective cohort study. Front Oncol.

[63] Tettey M, Edwin F, Aniteye E (2004). The changing epidemiology of oesophageal cancer in Sub-Saharan Africa-the case of Ghana. Pan Afr Med J.

[64] Alidina A, Gaffar A, Hussain F (2004). Survival data and prognostic factors seen in Pakistani patients with oesophageal cancer. Ann Oncol.

[65] Pedram A, Mahmodlou R, Enshayi A (2011). Oesophageal cancer in northwestern Iran. Indian J Cancer.

[66] Cherian JV, Sivavaman R, Muthusamy AK (2007). Carcinoma of the oesophagus in Tamil Nadu (South India):16 year trends from a tertiary center. J Gastrointestin Liver Dis.

[67] Pun CB, Aryal G, Basyal R (2012). Histological pattern of oesophageal cancer at BP Koirala memorial cancer hospital in Nepal: a three year retrospective study. J Pathol Nepal.

[68] Aledavood A, Anvari K, Sabouri G (2011). Oesophageal cancer in northeast of Iran. Iran J Cancer Prev.

[69] Mabula DM, Rambau PF, Chalya PL (2013). Endoscopic and clinicopathological patterns of oesophageal cancer in Tanzania: experiences from two tertiary health institutions. World J Surg Onc.

[70] Asombang AW, Kasongo N, Muyutu J (2021). Descriptive analysis of oesophageal cancer in Zambia using the cancer disease hospital database: young age, late stage at presentation. Pan Afr Med J.

[71] Jiang YX, Zhang DW, Chen Y (2015). The characteristics of oesophageal squamous cell carcinoma: an analysis of 1317 cases in southeastern China. Contemp Oncol (Pozn).

[72] Song T, Wan Q, Yu W (2017). Pretreatment nutritional risk scores and performance status are prognostic factors in oesophageal cancer patients treated with definitive chemoradiotherapy. Oncotarget.

[73] Nam SY, Jeon SW, Lee SJ (2020). Clinical factors to predict the response to concurrent chemoradiotherapy and survival in oesophageal cancer patients. Gut and Liver.

[74] Mmbaga EJ, Deardorff KV, Mushi B (2018). Characteristics of oesophageal cancer cases in Tanzania. J Glob Oncol.

[75] Patel K, Wakhisi J, Mining S (2013). Oesophageal cancer, the topmost cancer at MTRH in the Rift Valley, Kenya, and its potential risk factors. ISRN Oncol.

[76] Dawsey SP, Tonui S, Parker RK (2010). Oesophageal cancer in young people: a case series of 109 cases and review of the literature. PLoS One.

[77] Odera JO, Odera E, Githang J (2017). Oesophageal cancer in Kenya. Am J Dig Dis.

[78] Ocama P, Kagimu MM, Odida M (2008). Factors associated with carcinoma of the oesophagus at Mulago Hospital, Uganda. Afr Health Sci.

[79] Alema ON, Iva B (2014). Cancer of the oesophagus: histopathological sub-types in northern Uganda. Afr Health Sci.

[80] Mlombe YB, Rosenberg NE, Wolf LL (2015). Environmental risk factors for oesophageal cancer in Malawi: a case-control study Malawi. Med J.

[81] Come J, Castro C, Morais A (2018). Clinical and pathologic profiles of oesophageal cancer in Mozambique: a study of consecutive patients admitted to Maputo Central Hospital. J Glob Oncol.

[82] Ferndale L, Aldous C (2022). Oesophageal cancer in South Africa: a scoping review. S Afr J Oncol.

[83] Liang H, Fan JH, Qiao YL (2017). Epidemiology, etiology, and prevention of oesophageal squamous cell carcinoma in China. Cancer Biol Med.

[84] National Health Commission of the People's Republic of China (2019). Chinese guidelines for diagnosis and treatment of oesophageal carcinoma. Chin J Cancer Res.

[85] Opstelten JL, Wijkerslooth LR, Leenders M (2017). Variation in palliative care of oesophageal cancer in clinical practice: factors associated with treatment decisions. Dis Oesophagus.

[86] Ansari F, Rai A (2021). A retrospective study of the epidemiology of oesophageal cancer and its management in a Central Indian Institute. Int Surg J.

[87] Then EO, Lopeza M, Saleem S (2020). Oesophageal cancer: an updated surveillance epidemiology and end results database analysis. World J Oncol.

[88] Nassri A, Zhu H, Muftah M (2018). Epidemiology and survival of oesophageal cancer patients in an American cohort. Cureus.

[89] Kim S, DiPeri TP, Guan M (2020). Impact of palliative therapies in metastatic oesophageal cancer patients not receiving chemotherapy. World J Gastrointest Surg.

[90] Seraphin M, Silberstein PT, Cichon G (2022). Trends in palliative care interventions for stage IV oesophageal cancer: an analysis of the NCDB. J Clin Oncol.

[91] Wondimagegnehu A, Hirpa S, Abaya SW (2020). Oesophageal cancer magnitude and presentation in Ethiopia 2012–17. PLoS One.

[92] Cruz AIBM, Pinto LFR, Thuler LCS (2018). Profile of patients with oesophageal cancer diagnosed between 2001 and 2010 in Brazil. Rev Bras Cancerol.

[93] Nakimuli E, Ssentongo J, Mwaka AD (2021). Understanding health-seeking and adherence to treatment by patients with oesophageal cancer at the Uganda cancer Institute: a qualitative study. BMC Health Serv Res.

[94] Ambroggi M, Biasini C, Del Giovane C (2015). Distance as a barrier to cancer diagnosis and treatment: review of the literature. Oncologist.

